# Anthropometric measurements of the abdominal wall: toward standardization and difficulty grading in rTAPP inguinal

**DOI:** 10.3389/jaws.2026.16789

**Published:** 2026-06-18

**Authors:** U. A. Dietz, R. Frey, A. Lalos, U. Pfefferkorn, M. Ramser

**Affiliations:** 1 Department of Visceral, Vascular and Thoracic Surgery, Cantonal Hospital Olten, Olten, Switzerland; 2 Department of Visceral and Transplantation Surgery, University Hospital of Zurich, Zurich, Switzerland

**Keywords:** abdominal elevation angle, abdominal wall compliance, rTAPP inguinal, anisotropy, groin hernia

## Abstract

**Introduction:**

Robotic-assisted procedures in hernia surgery are highly standardized, whereas the distance between the ports and the target organ is of critical importance. To date, anthropometric data regarding the distance from the umbilicus to the symphysis and the Abdominal Elevation Angle (AEA) are lacking, both of which may be relevant for preoperative estimation of procedural difficulty and for the standardization of the surgical technique. This study prospectively evaluated anthropometric abdominal wall parameters and their influence on procedural difficulty in robotic-assisted groin hernia repair.

**Methods:**

Abdominal wall elasticity was assessed by measuring xiphopubic, xiphoumbilical, umbilicopubic, and transversal distances before and after pneumoperitoneum at 12 mmHg. The abdominal elevation angle (AEA) was measured photographically and categorized as narrow (<23.8°), normal (23.9°–40.0°), or wide (>40.1°). Intraoperative anatomical features including intra-abdominal fat, peritoneal thickness, and adhesions were recorded using Likert scales.

**Results:**

Sixty-eight consecutive patients (61 male, 7 female; mean age 65 years, mean BMI 25.3 kg/m^2^) undergoing elective robotic-assisted groin hernia repair were included between July 2020 and April 2021. A total of 96 hernia sides were operated, including 28 bilateral and 8 recurrent cases. All distances increased significantly under pneumoperitoneum (p < 0.0001); the mean umbilicopubic distance with pneumoperitoneum was 16.35 cm. Mean longitudinal elasticity was 13.74% and transversal elasticity 11.73%, with no statistically significant difference between the two directions (p = 0.0844). Neither AEA nor BMI showed a statistically significant correlation with procedural difficulty. However, multiple linear regression analysis identified longitudinal abdominal wall elasticity as an independent predictor of procedural difficulty (p = 0.0139). Surgeon satisfaction with the procedural outcome was consistently high across all patient groups (mean score of 9.76/10), regardless of BMI or AEA.

**Conclusion:**

The mean umbilicopubic distance is less than the 20 cm proposed as distance-to-target in robot-assisted procedures; the umbilicus is not ideal for positioning of the endoscope port. Longitudinal abdominal wall elasticity appears to be a relevant factor influencing the difficulty of robot-assisted groin hernia repair, whereas BMI and AEA alone are insufficient predictors.

## Introduction

Over the past 30 years, minimally invasive techniques for inguinal hernia repair (TEP and TAPP) have become established due to their low morbidity (recurrence rates comparable to open procedures and less chronic pain than open procedures) [1, 2]. To further improve these outcomes, robot-assisted surgical systems are now widely available. The surgical instruments of robotic systems have complex joints and require a standardized distance from the internal inguinal region; a distance of 20 cm between the ports and the symphysis has proven to be a practical working distance, which means, however, that the camera port is not necessarily positioned at the level of the umbilicus [3, 4]. This represents a departure from the classic aesthetic principles of minimally invasive surgery, where the umbilicus is preferred for camera insertion because of the concealed scar. One of the distinctive features of robot-assisted surgery is its high degree of standardization; the optimal visibility, the instruments’ degrees of freedom, and the ergonomics provide the flexibility to perform the operation precisely even under challenging conditions (e.g., obesity) using adjuvant interventions such as dorsal mesh fixation, suture closure of the internal inguinal ring, or suturing of the medial hernia with visible nerve dissection [3].

Several questions remain to be clarified:

- What is the distance from the umbilicus to the symphysis in the uninflated state and under 12 mmHg pneumoperitoneum pressure?
- What is the elasticity of the abdominal wall in the longitudinal and transverse directions under 12 mmHg pneumoperitoneum? Is the postulated anisotropy of the abdominal wall reproducible under the study conditions?
- What is the range of the Abdominal Elevation Angle (AEA)?
- In robot-assisted rTAPP, do the AEA and BMI influence the complexity of the procedure and the surgeon’s satisfaction with the outcome?
- Are preoperative anthropometric measurements useful in predicting the degree of difficulty?

## Methods

Consecutive patients planned for elective robot-assisted groin hernia repair and assigned to be operated by two of the authors (UAD and MR) in the period from July 2020 to April 2021 were eligible. Demographic data were extracted from the clinical information system. Eligibility criteria were absence of abdominal scars from previous open surgery and age >18 years. Patients consented according to the General Consent Form (Ethics Committee of Northwestern Switzerland, approval number 2020-00525). The methodology of the surgical procedure followed published protocols [3, 4]. BMI was classified according to WHO: <30.0 kg/m^2^ (normal), 30.0–34.9 kg/m^2^ (grade I), 35.0–39.9 kg/m^2^ (grade II), >40.9 kg/m^2^ (grade III).

### Native anthropometric measurements(pre-intervention)

A 10 cm line was marked on the sterile drape on the patient’s right side using a ruler as a reference. Before starting the procedure, the following distances were measured with the sterile ruler and noted (first measurement): xiphoumbilical distance and umbilicopubic distance (the xiphopubic distance was calculated later from the sum of both), as well as the transverse distance (from the median axillary line on the right side to the corresponding line of the left side, at the level of the umbilicus). The exact measurement points were marked on the skin with a pen in order to later take the second measurement, with pneumoperitoneum (see below).

### Anthropometric measurements under pneumoperitoneum (post-intervention)

Pneumoperitoneum was created using a Veres needle at Palmer’s point. After reaching the pneumoperitoneum pressure of 12 mmHg, the same distances were measured again and recorded (see above). The difference between the post-interventional and pre-interventional measurements was used to calculate the elongation or elasticity of the abdominal wall in the longitudinal and transverse directions as a percentage.

### Measurement of the abdominal elevation angle (AEA)

**FIGURE 1:**
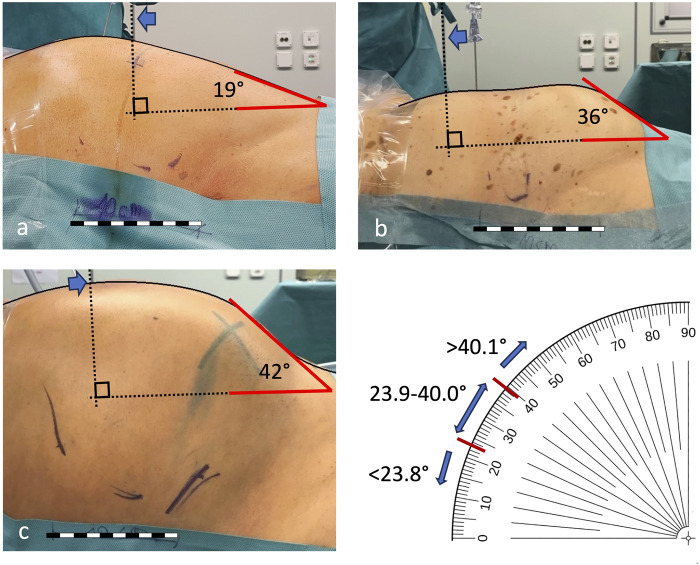
Calculation of the abdominal elevation angle (AEA, red) at 12 mmHg of pneumoperitoneum pressure. **(a)** = narrow angle (limited elevation of the abdominal wall); **(b)** = main normal angle; **(c)** = wide angle (significant elevation of the abdominal wall). Blue arrow: vertical line at the OR-Wall (orientation of the 90° angle of the triangle). Dotted lines correspond to an imaginary right-angled triangle; measuring stick with 10 cm in length.

The abdominal elevation angle was documented with photographs taken from the right side of the patient. The camera was positioned at a 90° angle to the right side of the patient and horizontally to the floor. To maintain the horizon (measure to prevent bias, *sine qua non* condition for measuring the angle), the operating table was secured in horizontal position (0°) and the vertical mark on the opposite wall of the operating room was targeted (Figure 1, blue arrows). The angles were evaluated in a standardized manner based on the photographic documentation. Angles <23.8° were considered narrow, angles between 23.9° and 40.0° were considered normal and angles >40.1° were considered wide (Figure 1). Following the photographic documentation, the trocars were inserted, the ports positioned, and the intra-abdominal pressure was reduced to 8 mmHg for the actual operation.

### Anatomical features

To record anatomical features that could influence the degree of difficulty of the operation, three criteria were assessed by the surgeon using Likert scales. a) Intra-abdominal fat was classified as slim (1), medium (2), fatty (3), or very fatty (4); b) The thickness of the peritoneum was classified as very thin (1), normal (2), or strong (3); and c) Adhesions along the inferior epigastric vessels were classified as none (1), mild (2), or severe (3).

### Surgeon satisfaction with the final result

At the end of the operation, the surgeon rated the final result on a scale of 1–10 in the categories of procedural difficulty (from low 1 to high 10) and satisfaction with the procedural outcome (from low 1 to high 10).

### Statistics

Descriptive statistics with mean, median, minimum, maximum, and standard deviation were calculated as appropriate to analyze the distribution in each category; normality tests were performed to validate the generalizability of the data (Shapiro-Wilk test). To compare continuous data without and with pneumoperitoneum, the paired two-tailed t-test with Welch’s correction was applied. Ordinally scaled variables (Likert scale) were summarized using medians and interquartile ranges. Additionally, the distribution of responses across categories was presented as percentages. To determine the grade of difficulty and satisfaction, a one-way ANOVA was applied. To analyze if BMI, abdominal elevation angle, abdominal volume and abdominal wall elasticity had an influence on the degree of difficulty, a multiple linear regression analysis was performed. Graphs are presented as box and whisker plots or estimation plots, showing the individual paired values and mean differences. GraphPad Prism Version 10.6.1 was used.

No funding was needed for this study.

## Results

The study included 68 consecutive patients (61 male and 7 female); in total, 96 hernia sides were operated, with 28 bilateral hernias and 8 recurrent sides. On average, 4 L of CO_2_ were required to achieve a pressure of 12 mmHg (range 2.8–6.8 L) (Table 1).

**TABLE 1:** Demography.

Demography	Mean (SD)	Range
Age, years (range)	65	18–92
Height (cm)	175	156–192
Weight (kg)	70	53–121
BMI (kg/m^2^)	25.3	20.10–39.50
Abdominal volume (liters of CO_2_)	4	2.3–6.8
​	N	%
Male sex/total patients	61/68	89.70
Female sex/total patients	7/68	10.29
Recurrent sides/total hernia sides	8/96	8.33
Previous abdominal surgery/total patients	10/68	14.70

### Anthropometric measurements

#### Xiphopubic distance (cm) without and with PP

**FIGURE 2:**
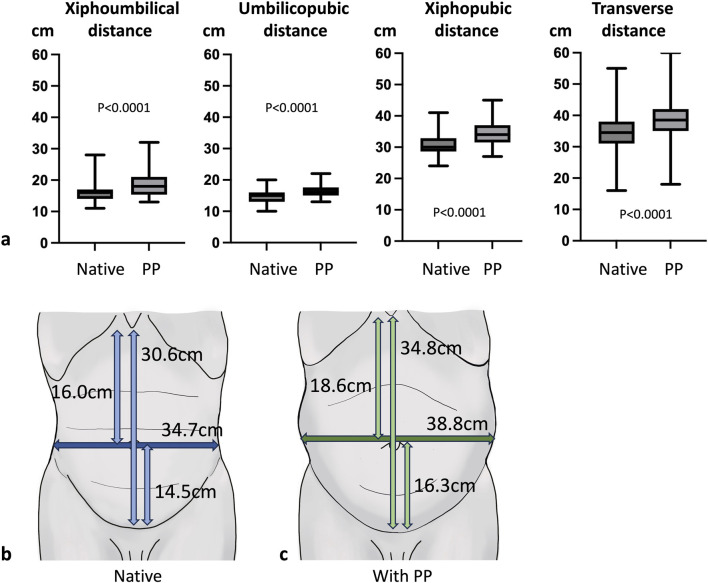
Anthropometric measurements of the abdominal wall elasticity. **(a)** Box and whisker-plots with median and interquartile values (25%–75%) as well as whiskers on minimum and maximum; each graph shows the normal values (first measurement) and the distance under pneumoperitoneum (second measurement) for each of the segments (Paired t-test). **(b)** Longitudinal and transversal measurements without pneumoperitoneum. **(c)** Longitudinal and transversal measurements without pneumoperitoneum (blue arrows = native measurements; green arrows = with 12 mm pneumoperitoneum pressure).

The mean xiphopubic distance was 30.59 cm without, compared to 34.74 cm with pneumoperitoneum. The median values were 30.00 cm vs. 34.00 cm, respectively, corresponding to 13.80% of elasticity. These measurements passed the normality test based on the SD (Shapiro-Wilk test) with p value 0.0974 (yes) and 0.0784 (yes), respectively. The increase in distance after pneumoperitoneum was significant (paired t-test, two tailed: p < 0.0001, significant) (Figure 2).

#### Xiphoumbilical distance (cm) without and with PP

The mean xiphoumbilical distance was 15.99 cm without, compared to 18.43 cm with pneumoperitoneum. The median value was 16.00 cm vs. 18.00 cm, respectively, corresponding to 15.92% of elasticity. These values failed to pass the normality test based on the SD (Shapiro-Wilk test) with p value 0.0001 (no) and 0.0008 (no), respectively. Nevertheless, the increase of the xiphoumbilical distance after pneumoperitoneum was significant (paired t-test, two tailed, p < 0.0001) (Figure 2).

#### Umbilicopubic distance (cm) without and with PP

The mean umbilicopubic distance was 14.66 cm without, compared to 16.35 cm with pneumoperitoneum. The median values were 15.00 cm vs. 16.25 cm respectively, corresponding to 12.13% of elasticity. These measurements passed the normality test based on the SD (Shapiro-Wilk test) with p value 0.9701 (yes) and 0.9731 (yes), respectively. The increase in distance after pneumoperitoneum was significant (paired t-test, two tailed, p < 0.0001) (Figure 2). The xiphoumbilical elasticity was significantly higher than the umbilicopubic elasticity (unpaired t-test with Welch’s correction, 2-tailed, p = 0.0202).

#### Transversal distance (cm) without and with PP

The mean transversal distance was 34.48 cm without, compared to 38.53 cm with pneumoperitoneum. The median was 34.40 cm vs. 38.50cm, respectively. These values passed the normality test based on the SD (Shapiro-Wilk test) with p value 0.9707 (yes) and 0.9726 (yes), respectively. The increase in distance of the transversal distance is significant (paired t-test, two tailed, p < 0.0001) (Figure 2).

#### Anisotropy, or the comparison of longitudinal and transversal elasticity (%)

**FIGURE 3:**
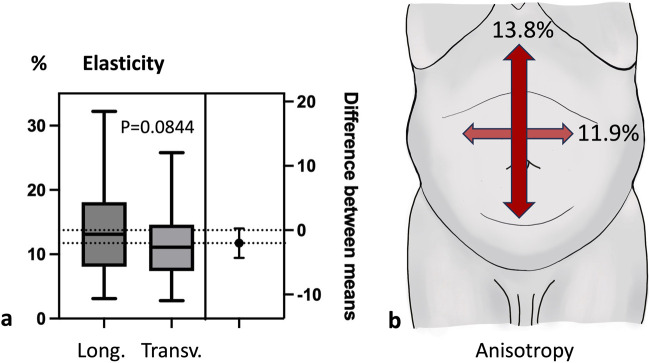
Anisotropy. **(a)** Estimation plot (Gardner-Altman) with median and interquartile values (25%–75%), whiskers on minimum and maximum; the dotted lines represent 95% confidence interval (95% CI) of the difference between the means; **(b)** The abdominal wall is more elastic in longitudinal than in transversal direction (not statistically significant in the current series). Unpaired t-test with Welch’s correction, non-significant. Long. = longitudinal; Trans. = transversal.

The mean elasticity was 13.74% in the longitudinal and 11.73% in the transversal direction. The median elasticity was 13.10% vs. 11.10%, respectively. There is no statistical difference between the elasticity of the longitudinal compared to the transversal distance, despite the higher values for the longitudinal distance (unpaired t-test with Welch’s correction, p = 0.0844) (Figure 3).

### Measurement of the abdominal elevation angle (AEA)

The measured AEA passed the normality test of Shapiro-Wilk (p = 0.3637). For evaluation of the influence of the AEA on the grade of difficulty and the grade of satisfaction, patients were divided into 3 groups according to angle: a) mean normal angle (mean plus minus one SD, i.e., range from 23.9 to 40.0; n = 53), b) narrow angle (smaller than normal, i.e., <23.8; n = 8) and c) wide (greater than normal, i.e., >40.1; n = 7). The values for the AEA are shown in Table 2.

**TABLE 2:** Abdominal elevation angle after pneumoperitoneum.

AEA	Male (n = 61)	Female (n = 7)
Minimum	17.72°	27.59°
Maximum	51.96°	42.21°
Mean	**31.95°**	**34.68°**
Median	32.18°	34.03°
Range	34.24°	14.61°
SD	8.072	5.345
95% CI of mean	29.50–34.41	29.07–40.28

Male vs. female p = 0.2715. AEA, Abdominal elevation angle.

### Anatomical special features

In terms of intra-abdominal fat, 12 patients (17.9%) were slim, 23 (34.3%) medium, 18 (26.8%) fatty, and 9 (13.4%) very fatty. The thickness of the peritoneum was described as thin in 17 hernia sites (25.3%), normal in 43 (64.1%), and strong in 6 (8.9%). Adhesions of the tissue in the area surrounding the inferior epigastric vessels and in the space of Retzius occurred mostly in cases of recurrence and especially after prostatectomy; there were no adhesions in 13 hernia sites (19.4%), adhesions were minor in 35 (52.2%), and adhesions were pronounced in 18 hernia sites (26.8%); no information was available for 2 hernia sites (2.9%) (Table 3).

**TABLE 3:** Intraoperative findings.

​	Mean	Range	n	%
Intraabdominal fat (Likert 1-4)	2.27	1–4	​	​
Thickness of peritoneum (Likert 1-3)	1.84	1–3	​	​
Adhesions to inf. epigastric vessels (Likert 1-3)	2.07	1–3	​	​
Varices of spermatic vessels (n)	​	​	8	11.76
Length of hernia sac (>2 cm)	​	​	40	58.80
*In toto* mobilization of inner sac (n)	​	​	63	92.64

### Procedural grade of difficulty

The abdominal elevation angle (AEA) did not correlate with the degree of difficulty of the operation (p = 0.1289), although a tendency toward less difficulty was calculated in patients with mean to normal angles (23.9°–40.0°) (Table 4). Similarly, the grade of difficulty also did not correlate with the BMI (p = 0.0930) (Table 5).

**TABLE 4:** Grade of difficulty (1-10) according to the abdominal elevation angle.

AEA	All	Wide	Mean	Narrow
​	(n = 68)	(n = 7)	(n = 53)	(n = 8)
Minimum	2.00	4.00	2.00	2.00
Maximum	10.00	10.00	10.00	10.00
Mean	6.25	7.66	6.05	6.83
Median	6.00	8.00	6.00	7.50

1.00 = lowest to 10.00 = highest grade of difficulty.

Ordinary one-way ANOVA, p = 0.1289.

**TABLE 5:** Grade of difficulty (1-10) according to the BMI.

BMI	All	Normal	Grade I	Grade II
​	(n = 68)	(n = 62)	(n = 4)	(n = 2)
Minimum	2.00	2.00	3.00	7.00
Maximum	10.00	10.00	8.00	10.00
Mean	6.25	6.16	6.75	8.50
Median	6.00	6.00	8.00	8.50

1.00 = lowest to 10.00 = highest grade of difficulty.

Ordinary one-way ANOVA, p = 0.0930.

### Surgeon satisfaction with the final result

Despite the difficulty of the operations (as shown in the previous table), the degree of procedural satisfaction at the end of the operation was very high regardless of the angle (Tables 6, 7).

**TABLE 6:** Grade of procedural satisfaction (1-10) according to the abdominal elevation angle.

AEA	All	Wide	Mean	Narrow
​	(n = 68)	(n = 7)	(n = 53)	(n = 8)
Minimum	8.00	9.00	8.00	9.00
Maximum	10.00	10.00	10.00	10.00
Mean	10.00	9.66	9.76	9.83
Median	9.76	10.00	10.00	10.00

1.00 = lowest to 10.00 = highest grade of satisfaction.

One-way ANOVA, non-parametric, assuming Gaussian distribution, p = 0.7654.

**TABLE 7:** Grade of procedural satisfaction (1-10) according to the BMI.

BMI	All	Normal	Grade I	Grade II
​	(n = 68)	(n = 62)	(n = 4)	(n = 2)
Minimum	8.00	8.00	10.00	9.00
Maximum	10.00	10.00	10.00	10.00
Mean	10.00	9.75	10.00	9.50
Median	9.76	10.00	10.00	9.50

1.00 = lowest to 10.00 = highest grade of satisfaction.

Non-significant.

### Multivariate analysis

Multiple linear regression, analyzing the subpopulation of unilateral hernias, showed a significant effect of the longitudinal elasticity on the procedural difficulty [F (1, 22) = 7.144, p = 0.0139]. BMI, transversal elasticity, abdominal CO_2_ volume and abdominal elevation angle were not independent factors for increased procedural difficulty.

## Discussion

The elasticity of the abdominal wall is a basic prerequisite for minimally invasive surgery with pneumoperitoneum. The expansion of the abdominal cavity (abdominal compliance, C_ab_) under pneumoperitoneum is the result of the elasticity of the abdominal wall and diaphragm. Abdominal compliance has limits and cannot be expanded arbitrarily by increasing pressure or neuromuscular relaxation [5]. Intra-abdominal pressure initially increases linearly with the volume of pneumoperitoneum until the limit of C_ab_ is reached [6]. Conversely, however, sufficient intra-abdominal working space can be gained at low pneumoperitoneum pressure by lifting the abdominal wall (lift-laparoscopy), which is also standard practice with robotic arms [7]. It is also known that the abdominal wall of women is more elastic than that of men (i.e., it has a lower Young’s modulus or is more compliant), which may be due to hormonal factors or at least related to the phenomenon of “pre-stretching,” e.g., after pregnancy [8]. The type of positioning on the operating table can also have an influence on the C_ab_, especially in the anti-Trendelenburg position, but this does not apply to the patients in our study cohort, who are operated on in the Trendelenburg position [9]. According to clinical experience, abdominal scars, previous operations, previous pregnancies, intra-abdominal adhesions, CAPD, or ascites have an influence on the C_ab_ and may be used as predictive information for estimating in advance the grade of difficulty. In the context of robot-assisted transversus abdominis release (rTAR), the abdominal wall is weakened by detachment of the medial insertion of the transversus abdominis (MTA), which leads to an intervention-related higher compliance, as is also the case with botulinum toxin (Botox), for example.

The type of abdominal fat distribution is described as a predictive factor for the elasticity of the abdominal wall: with gynoid fat distribution, the abdominal wall has an oval shape transversely and can therefore theoretically be stretched better using pneumoperitoneum than the already round shape of the abdominal wall with android fat distribution [5]. However, experience shows that with android fat distribution, the abdominal wall is usually thin, which in turn increases its elasticity (low Young’s modulus), while a gynoid thick subcutaneous fat layer requires high pneumoperitoneum pressure to create sufficient intra-abdominal space, as demonstrated before [10].

The elasticity of the abdominal wall is higher in the longitudinal than in transversal direction [5, 11, 12]. According to our results, the supraumbilical longitudinal elasticity is significantly higher than the infraumbilical elasticity (15.92% vs. 12.13%, respectively, p = 0.0202); in the subgroup analysis of women, no significant difference was found; explanations for this difference may be the small number of female patients, but probably the overstretching of the abdominal wall as a whole during pregnancy may contribute to increased infraumbilical elasticity also. Why the abdominal wall is more elastic supraumbilically than infraumbilically in men is not clear, maybe it is related to the higher prevalence of type I rectus diastasis according to Corvino in males, but this was not investigated in the current study [13]. Another explanation may be related to the different anatomical configuration of the supraumbilical linea alba, due to the physiological umbilical hernia, due to the physiological umbilical defect in the eighth embryonic week, which arises supraumbilically, while the embryonal Linea alba remains closed in the infraumbilical region to protect the urinary bladder [14]. Probably the infraumbilical region must be considered as a distinct part of the abdominal core. Other possible explanations for the longitudinal elasticity are the muscular anatomy (the rectus muscles are easier to stretch than the complex lateral abdominal wall) and the cylindrical shape of the abdominal cavity (tangential tension is expected to be higher than longitudinal tension) [5, 11, 12]. From a statistical point of view, nevertheless, measurements of the supraumbilical distances failed to pass the normality test based on the standard deviation (Shapiro-Wilk test, inconsistent with a Gaussian distribution); probably a larger population would be needed to achieve a smaller standard deviation, but since the xiphoumbilical length after pneumoperitoneum is secondary for the positioning of the ports (the distance to target is measured from the pubic bone), this result is not relevant in the context of rTAPP.

The infraumbilical (umbilicopubic) distance after pneumoperitoneum is–according to the present measurements–not sufficient for an optimal positioning of the ports at the umbilicus, which should be aimed at 20 cm distance to target; as a consequence, the umbilicus is not useful as orientation for port placement; according to our results, in more than 50% of patients (median of 16.25 cm), the port for the endoscope should be placed cranially to the umbilicus and ideally slightly lateral to the linea alba. For the sake of completeness, it should be noted that due to the longitudinal elasticity of the abdominal wall, the port position in rTAPP is to be marked with a ruler after reaching the pressure of 12 mmHg pneumoperitoneum.

The transversal elasticity after pneumoperitoneum (11.90%) is less than the longitudinal elasticity (13.80%) (Figure 3); as a consequence, the distribution of the intraabdominal pressure is higher in the transverse direction than in the longitudinal direction [7, 8, 11]. This difference in elasticity or compliance of the abdominal wall in the longitudinal and transverse directions is called anisotropy. Whereas the anisotropy may not be relevant in rTAPP, it needs to be considered for closing defects of the Linea alba and for choosing the proper mesh in ventral hernia repair. For example, closing an umbilical hernia with 4 cm diameter in transverse fashion protects the suture line from tension; closing it in longitudinal way, exposes the scar to the higher radial tension forces [12, 15]. Furthermore, studies show that surgical meshes with anisotropy comparable to the abdominal wall integrate better because they match the natural tension conditions of the abdominal wall [16] and the anisotropy of the abdominal wall needs also to be taken into consideration not only when implanting meshes but also in the process of designing new meshes [15, 17]. For the TARUP procedure, other anthropometric data would be important: the Carbonell formula can be calculated from the CT images, and the visceral fat tissue (android or gynoid fat distribution) and thickness of the musculature of the abdominal wall could likely provide clues regarding the AEA or abdominal wall compliance, thereby optimizing port placement and, if necessary, helping to choose between a caudal and a lateral approach [18]. The umbilical position is not relevant for TARUP.

To our knowledge, the abdominal elevation angle (AEA) was not previously described in the literature. The median difficulty score was 6.00 in the group of patients with an abdominal elevation angle of 20°–39°; although the median was higher at >40° (8.00) and <19° (7.50), this was not statistically significant (p = 0.1289). A similar, non-significant result was also found for BMI (p = 0.0930). Although from a subjective point of view both a narrow and a wide abdominal elevation angle appear to make the endoscopic operation of groin hernia more difficult, this hypothesis could not be confirmed in the current study. In the one-way ANOVA statistics, the abdominal elevation angle did not correlate with the degree of difficulty of the procedure (p = 0.1289). One possible explanation for this is the small number of patients in the outlying groups (11 with narrow or wide AEA and 6 with obesity grade I and II), which is too small to be statistically significant. Another explanation may be due to the optimal working conditions of the robotic platform, which, thanks to the degrees of freedom of the instruments, facilitates working at angles that are too narrow or too wide and allows optimal access to the anatomical structures of the abdominal wall, compensating for the difficulties associated with obesity. A further explanation may be the high level of experience of the surgeons involved. A comparative study between conventional laparoscopy and robot-assisted surgery would possibly reveal a difference here, although this will likely not be performed.

Despite the difficulty of the operations (as shown in the previous table), the degree of procedural satisfaction at the end of the operation was very high regardless of the angle (Tables 6, 7). The grade of satisfaction is based on the final result of the treatment of the hernia orifices and the anatomical preparation of the myopectineal orifice as well as the creation of the landing zone for the mesh. Despite the anisotropy of the abdominal wall and the different grades of difficulty due to the abdominal elevation angle and the BMI, the robotic platform enabled the surgeons to accomplish the expected results. Since the positioning of the ports was standardized at 20 cm to the pubic bone, port positioning may have contributed to this result also. Multiple linear regression showed a significant effect of the longitudinal elasticity on the procedural difficulty (high sum of squares of 18.97 and high F of 7.144, p = 0.0139). BMI, transversal elasticity, abdominal CO_2_ volume and abdominal elevation angle were not independent factors for increased procedural difficulty, probably due to the low number of included patients.

AEA, abdominal wall elasticity, and insufflated CO_2_ volume are not surrogates for planning the expected duration of surgery, as they can only be measured intraoperatively. Further studies are needed to generate a reliable and useful preoperative predictive score, for optimization of OR capacity as soon as during preoperative consent consultation [19].

The current study has several limitations: differences between males and females could not be confirmed due to the small number of patients; the decisive factor for the lower proportion of women is that the incidence of inguinal hernia in women is 10-fold lower than in men. Another limitation is the computation of the operating time, which is influenced by many independent variables: expertise, complexity of the case, anatomy of the hernia, concomitant findings, recurrences, unilateral or bilateral, previous operations such as prostatectomy, age (soft or stiff tissue), time lost due to contemplation of the amazing anatomy, and many others. The surgical team involved had experience with at least 500 rTAPPs (experience may be a source of bias). Therefore, operating time was not used to assess difficulty in the current study. To group the AEA following the mean and SD was used to compare the extremes in relation to the norm; the main limitation of this approach is the greater number of patients in the normal group in comparison with the outlier groups (narrow and wide AEAs); this may be a bias in the interpretation of the results; otherwise to create groups with comparable number of patients for the AEA, an algorithm to reduce the number of the normal population (exclusion criteria for the “some of the normals”) would be necessary, creating another source of bias.

In conclusion, the mean distance from the umbilicus to the pubic bone under 12 mmHg pneumoperitoneum pressure is less than the 20 cm port-to-target-organ distance recommended for rTAPP; the Abdominal Elevation Angle (AEA) under 12 mmHg of pneumoperitoneum pressure in males had a wide range, from 17.72° to 51.96°; and finally, neither AEA nor BMI showed a statistically significant correlation with procedural difficulty. However, multiple linear regression analysis identified longitudinal abdominal wall elasticity as an independent predictor of procedural difficulty (p = 0.0139). Our data failed to define preoperative anthropometric information useful to predict the degree of difficulty of the procedures.

The results of our study are partially generalizable, with the recommendation for port placement 20 cm cranial to the pubic bone instead of using the umbilicus as reference, the definition of the AEA and the anisotropy being the most relevant. The results aim to standardize the rTAPP and further refine clinical outcomes, but further prospective studies involving a larger number of patients and surgeons with different levels of experience are needed.

## Data Availability

The raw data supporting the conclusions of this article will be made available by the authors, without undue reservation.
